# Novel Agent Nitidine Chloride Induces Erythroid Differentiation and Apoptosis in CML Cells through c-Myc-miRNAs Axis

**DOI:** 10.1371/journal.pone.0116880

**Published:** 2015-02-03

**Authors:** Na Liu, Peng Li, Shaolei Zang, Qiang Liu, Daoxin Ma, Xiulian Sun, Chunyan Ji

**Affiliations:** 1 Department of Hematology, Qilu Hospital of Shandong University, Jinan, China; 2 Key Lab of Otolaryngology, Qilu Hospital of Shandong University, Jinan, China; Emory University, UNITED STATES

## Abstract

The proto-oncogene c-Myc plays critical roles in human malignancies including chronic myeloid leukemia (CML), suggesting that the discovery of specific agents targeting c-Myc would be extremely valuable for CML treatment. Nitidine Chloride (NC), a natural bioactive alkaloid, is suggested to possess anti-tumor effects. However, the function of NC in leukemia and the underlying molecular mechanisms have not been established. In this study, we found that NC induced erythroid differentiation, accompanied by increased expression of erythroid differentiation markers, e. g. α-, ε-, γ-globin, CD235a, CD71 and α-hemoglobin stabilizing protein (AHSP) in CML cells. We also observed that NC induced apoptosis and upregulated cleaved caspase-3 and Parp-1 in K562 cells. These effects were associated with concomitant attenuation of c-Myc. Our study showed that NC treatment in CML cells enhanced phosphorylation of Thr58 residue and subsequently accelerated degradation of c-Myc. A specific group of miRNAs, which had been reported to be activated by c-Myc, mediated biological functions of c-Myc. We found that most of these miRNAs, especially miR-17 and miR-20a showed strong decrement after NC treatment or c-Myc interference. Furthermore, overexpression of c-Myc or miR-17/20a alleviated NC induced differentiation and apoptosis in K562 cells. More importantly, NC enhanced the effects of imatinib in K562 and primary CML cells. We further found that even imatinib resistant CML cell line (K562/G01) and CML primary cells exhibited high sensitivity to NC, which showed potential possibility to overcome imatinib resistance. Taken together, our results clearly suggested that NC promoted erythroid differentiation and apoptosis through c-Myc-miRNAs regulatory axis, providing potential possibility to overcome imatinib resistance.

## Introduction

Chronic myeloid leukemia (CML) is a hematopoietic stem/progenitor cell disorder in which BCR-ABL oncoprotein leads to a progressive block of differentiation and increased genetic instability [1]. Tyrosine kinase inhibitors (TKIs), specifically inhibiting BCR-ABL fusion protein and triggering apoptosis and differentiation of CML cells, are used as first-line treatment for CML [2]. Although TKIs have revolutionized the treatment of CML, CML is rarely curative [3]. Exploring novel differentiation inducer is considered an alternative strategy for CML therapy.

The proto-oncogene c-Myc has been shown to play pivotal roles in cell cycle regulation, metabolism, apoptosis, differentiation, cell adhesion and tumorigenesis [4]. Study showed that BCR-ABL indirectly activated c-Myc via either Janus-activated kinase 2 (JAK2) pathway [5] or the mitogen-activated protein kinase (MAPK) pathway [6]. c-Myc expression was elevated in CML blast crisis and correlated with poor response to imatinib (IM) [7]. c-Myc antagonized imatinib or dasatinib induced erythroid differentiation [8] and apoptosis [9], suggesting its vital roles in drug sensitivity. An increasing body of work suggested that disease relapse upon cessation of TKI therapy could be due to CML stem cells, which were resistant or refractory to treatment [10]. c-Myc was overexpressed in CML CD34^+^ cells compared with normal CD34^+^ cells [11], and determined transcriptional profiles of ATP-binding cassette (ABC) transporter genes, leading to drug efflux and resistance in CML stem cells [12], which indicated the importance of c-Myc in maintaining leukemic stem cells. The vital functions of c-Myc in CML suggested that further mechanistic understanding of c-Myc and finding novel agents targeting c-Myc would be a promising strategy for the treatment of CML.

Nitidine Chloride (NC), derived from *Zanthoxylum nitidum*, had been identified as a potential anti-tumor drug in several tumors, e. g. breast cancer [13, 14], nasopharyngeal carcinoma [15], renal cancer [16, 17] and hepatocellular carcinoma [18]. These studies demonstrated that NC suppressed growth of various cancer cells *in vitro* or *in vivo* by causing G2/M cell cycle arrest through suppressing cyclin B1-and p53-dependent pathway [15, 16, 18]. NC was also reported to induce cell apoptosis of renal cancer cells via the ERK-associated signaling pathway, accompanied by upregulation of Bax and downregulation of Bcl-2 [16]. Furthermore, NC had been found to modulate cell migration and invasion in breast cancer and renal cancer cells through the c-Src-fak and AKT signaling pathway [14, 17]. Recently, accumulating evidences suggested that NC could regulate STAT3 and VEGF levels, which were critical factors involved in the process of tumor angiogenesis [19]. NC had also been proven to be a powerful chemosensitizer for tumors [13]. However, the function of NC in leukemia and the underlying molecular mechanisms have not been established.

In this study, we found that NC could induce erythroid differentiation and apoptosis. These effects were associated with concomitant attenuation of c-Myc. Our study showed that treatment of NC promoted c-Myc degradation via enhanced phosphorylation of Thr58 residue, probably independent of GSK3β. We also observed that a specific group of miRNAs (miR-17, miR-20a, miR-30a, miR-221, miR-222 and miR-378), which were activated by c-Myc and executed part of c-Myc functions in leukemia development [11, 20, 21, 22], was markedly downregulated. Furthermore, overexpression of c-Myc or miR-17/20a alleviated NC induced differentiation and apoptosis in K562 cells. More importantly, NC enhanced the effects of imatinib in K562 and primary CML cells. We further found that even imatinib-resistant CML cell line (K562/G01) and CML primary cells exhibited high sensitivity to NC, which showed potential possibility to overcome imatinib resistance. Taken together, our results clearly suggested that NC promoted erythroid differentiation and apoptosis through c-Myc-miRNAs regulatory axis, providing potential benefits in both imatinib-sensitive and -resistant CML patients.

## Materials and Methods

### Cell culture and experimental reagents

K562 and K562/G01 cell line purchased from Chinese Academy of Medical Sciences (Tianjin, China), were cultured in RPMI 1640 medium supplemented with 10% fetal bovine serum (Gibco, Grand Island, NY) and penicillin-streptomycin in an incubator maintained at 37°C in an atmosphere containing 5% CO_2_. NC was purchased from Tauto Biotech (Shanghai, China). Primary antibodies for c-Myc and p21 were obtained from Cell Signaling Technology (Beverly, MA). Primary antibody against c-Myc pThr58 was purchased from ImmunoWay (Newark, DE). Primary antibody against globin γ was purchased from Santa Cruz Biotechnology (Santa Cruz, CA). Primary antibody against β-actin was from Sigma-Aldrich (St Louis, MO). FITC-conjugated CD71 and PE-conjugated CD235a were from eBioscience (San Diego, CA). All secondary antibodies were obtained from LI-COR Biosciences (Lincoln, NE). Benzidine, GSK3β inhibitors LiCl or Bio, and all other chemicals were obtained from Sigma-Aldrich (St Louis, MO).

### CML samples

Bone marrow samples of 5 initially diagnosed CML patients were obtained after informed consent at the Qilu Hospital of Shandong University. Mononuclear cells were prepared using Ficoll-Hypaque (Sigma-Aldrich, St Louis, MO), according to the manufacturer’s protocol. All the study protocols involved with patients were approved by the Medical Ethics Committee of Qilu Hospital of Shandong University, Jinan, China, and written informed consents were obtained from all patients.

### Plasmids, lentiviral particles production and stable transfection

cDNA sequences containing human pri-miR-17 or pri-miR-20a units were cloned by PCR (pri-miR-17 forward primer: 5’-CGACGCGT TGTTAGAGTTTGAGGTGTTAATTC-3’; pri-miR-17 reverse primer: 5’-CCATCGAT CACTTAGGGCAGTAGATGCT-3’; pri-miR-20a forward primer: 5’-CGACGCGT AGTTGTGCAAATCTATGC-3’; pri-miR-17 reverse primer:5’-CCATCGAT TAACCATAGAACAGTGTTC-3’). The pri-miR-17 or pri-miR-20a PCR products were inserted into pLVTHM plasmid to generate pLV-miR-17 and pLV-miR-20a plasmid, which were used to produce lentiviral particles. To package LV-miR-17, LV-miR-20a or control lentiviral particles, Hek293T cells were co-transfected with a mixture of 10μg pLV-miR-17, pLV-miR-20a or pLVTHM, and 6.67μg psPAX2, 3.3μg pMD2.G utilizing Lipofectamine 2000 transfection reagent (Invitrogen, Carlsbad, CA) according to the manufacturer’s protocol. 48, 72 and 96hrs after transfection, supernatants were collected and then concentrated with PEG8000 (Sigma-Aldrich, St Louis, MO).

c-Myc shRNA vector and control plasmid were purchased from Genechem (Shanghai, China). For stable transfection screening, transfections were performed in 24-well culture plates using Lipofectamine 2000 transfection reagent (Invitrogen, Carlsbad, CA). 2 days after transfection, K562 cells were selected by 1μg/mL puromycin (Sigma-Aldrich, St Louis, MO). pEGFP-c-Myc plasmids were prepared as described previously [23] and were subcloned into pIRES2-EGFP plasmid to generate pIRES-c-Myc. The pIRES-c-Myc plasmid was transfected into K562 cells while pIRES was used as control. Stable transfectants were selected in medium containing 800μg/mL G418 (Sigma-Aldrich, St Louis, MO).

### Benzidine staining

The benzidine stock solution contained 0.2% (w/v) benzidine hydrochloride in 0.5M acetic acid. Cells (1 × 10^5^) were washed twice with ice-cold phosphate-buffered saline (PBS). The cell pellets were resuspended in ice-cold PBS (50 μl). The benzidine solution (10 μl) containing hydrogen peroxide (final concentration, 0.0012% v/v) was added and incubated for 10mins at room temperature. Benzidine-positive cells were examined by light microscope. At least 200 cells were counted in triplicate for each sample.

### Colony-forming Assay

Following treatment with NC (4 μM) and/or imatinib (0.2 μM) for 24 hours, untreated and drug-treated cells were washed in RPMI 1640. Then, one thousand cells under each condition were mixed in 0.3% low-melting agarose in RPMI 1640 supplemented with 10% FBS and plated on 0.5% agarose-coated 6-well tissue culture plates, which prevented attachment of cells to the plates. 10 days after cell inoculation, colonies were examined and photographed under a light microscope.

### CHX chase assay

K562 cells were prior treated with NC for 2 hrs. Then cells were treated with 150 μg/mL cycloheximide (Sigma-Aldrich, St Louis, MO) for 0, 20 and 40 mins. Cells were then harvested and analyzed by western blot. GFP tagged wild type or T58A mutant plasmids were transfected into K562 cells. 36 hrs after transfection, cells were prior treated with NC for 2 hrs and then treated with 150 μg/mL cycloheximide for 0, 1 and 2 hrs. Cells were then harvested and analyzed by western blot.

### miRNAs expression analysis

To determine the expression level of mature miRNAs (miR-17, miR-20a, miR-30a, miR-221, miR-222 and miR-378) in K562 cells, All-in-One miRNA qRT-PCR Detection Kit (GeneCopoeia, Rockville, MD) was used following manufacturer’s protocol. Briefly, the extracted RNA was reverse-transcribed in the presence of a poly-A polymerase with an oligo-deoxythymidine adaptor. Quantitative PCR was then carried out with SYBR-Green detection following manufacturer’s protocol (GeneCopoeia, Rockville, MD). Data was analyzed using Ct method and normalized by RNU6.

### Quantitative real-time RT-PCR

Total RNA was extracted from cells or patient samples using TRIzol reagent (Invitrogen, Carlsbad, CA). For reverse transcription, cDNA was synthesized from 1 μg of total RNA using M-MLV RTase cDNA Synthesis Kit (Takara, Japan). Real-time RT-PCR was carried out using an ABI 7900HT Fast Real-Time PCR system (Foster City, CA) and performed with SYBR-Green PCR Master Mix (Toyobo, Japan) in a 20 μl reaction volume. A comparative Ct method (2^-ΔΔCT^) was used to analyze the relative gene expression. *β-actin* was used as the internal control. The primers for real-time quantitative PCR were as follows: *c-Myc*-F, 5’-TCAAGAGGTGCCACGTCTCC-3’; *c-Myc*-R, 5’-TCTTGGCAGCAGGATAGTCCTT-3’; *globin ε*-F, 5’-ATGGTGCATTTTACTGCTGAGG-3’; *globin ε*-R, 5’-GGGAGACGACAGGTTTCCAAA-3’; *globin α-*F, 5’-CCACCAAGACCTACTTCCCG-3’; *globin α-*R, 5’-GCAGTGGCTTAGGAGCTTGA-3’; *globin γ-*F, 5’-GCTTCCTGGCAGAAGATGGT-3’; *globin γ-*R, 5’-TCTGCATCATGGGCAGTGAG-3’; *AHSP*-F, 5’-GGATCTCATTTCCGCAGGATTG-3’; *AHSP*-R, 5’-CTGCTGCCTGTAATAGTTGATGT-3’; *CD235a*-F, 5’-ACAACTTGCCCATCATTTCTCTG-3’; *CD235a*-R, 5’-TCAGTCGGCGAATACCGTAAG-3’; *β-actin*-F, 5’-CACTGTGTTGGCGTACAGGT-3’; *β-actin*-R, 5’-TCATCACCATTGGCAATGAG-3’.

### Immunoblotting

For immunoblotting analysis, cells were harvested and washed with ice-cold PBS and lysed by sonication in a modified RIPA buffer (Beyotime, Shanghai, China) in the presence of protease inhibitor cocktail (Beyotime, Shanghai, China). Protein samples were quantified with the Bio-Rad Dc protein assay kit (Bio-Rad, Richmond, CA). Whole-cell lysates were separated on 10% glycine SDS-PAGE gel. Detection and quantification were performed with the LI-COR Infrared imaging system (Lincoln, NE).

### Statistical analysis

All data were expressed as means ± S.E. from three independent experiments. Statistical analysis were carried out using the Student’s t-test with SPSS.17. P < 0.05 was considered statistically significant.

## Results

### NC induced erythroid differentiation in CML cells

**Fig 1:**
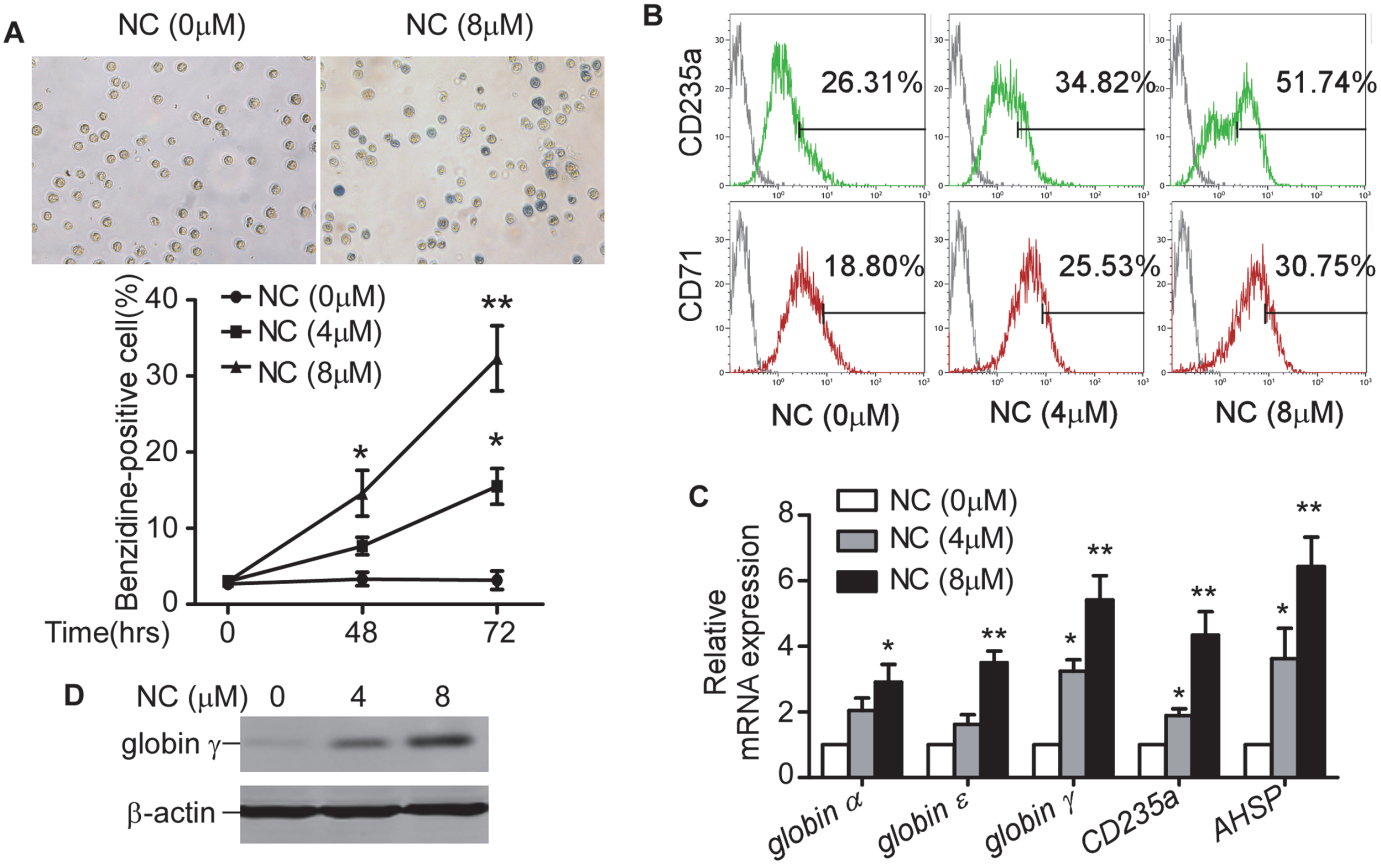
Nitidine Chloride induced erythroid differentiation in K562 cells. (A) K562 cells were treated with 0, 4, 8 μM NC for 48 or 72 hrs, and then benzidine staining was performed. A representative image of benzidine stained cells after treatment with NC for 72 hrs was shown. The percentage of benzidine-positive cells was calculated by counting at least 200 cells from 3 fields under microscope. The values represent the means ± S.E. (n = 3). *, *P*<0.05. **, *P*<0.01. (B) K562 cells were treated with 0, 4, 8 μM NC for 3 days, and then subjected to flow cytometry to determine the CD71 and CD235a positive cells. (C) Real-time qRT-PCR was used to analyze the gene expression of *globin α*, *globin ε*, *globin γ*, *CD235a* and *AHSP* in K562 cells after NC (4 or 8 μM) treatment for 2 days. *β-actin* was used as internal control. The values represent the means ± S.E. (n = 3). *, *P*< 0.05. **, *P*<0.01. (D) K562 cells were treated with 0, 4, 8 μM NC for 2 days. The expression of globin γ was determined by western blot.

After treatment with serial dosages of NC for 2 or 3 days, benzidine staining was performed to evaluate erythroid differentiation of K562 cells. Exposure to NC resulted in higher percentage of benzidine-positive cells in a dose- and time-dependent manner (Fig. 1A). We further found erythroid specific surface markers CD71 and CD235a were increased in K562 cells after NC treatment (Fig. 1B), which indicated that NC could induce erythroid differentiation in K562 cells. To confirm our findings, we evaluated the mRNA expression levels of erythroid differentiation markers *globin* (*globin α*, *ε and γ*), as well as *CD235a* and *α-hemoglobin stabilizing protein (AHSP)* by real-time qRT-PCR. Our results showed that all the erythroid differentiation markers mentioned above were significantly increased comparing with control group (Fig. 1C). The enhanced expression level of globin γ after NC treatment was further confirmed by western blot analysis (Fig. 1D). ALL these results indicated that NC could induce erythroid differentiation in K562 cells.

### NC induced apoptosis in CML cells

**Fig 2:**
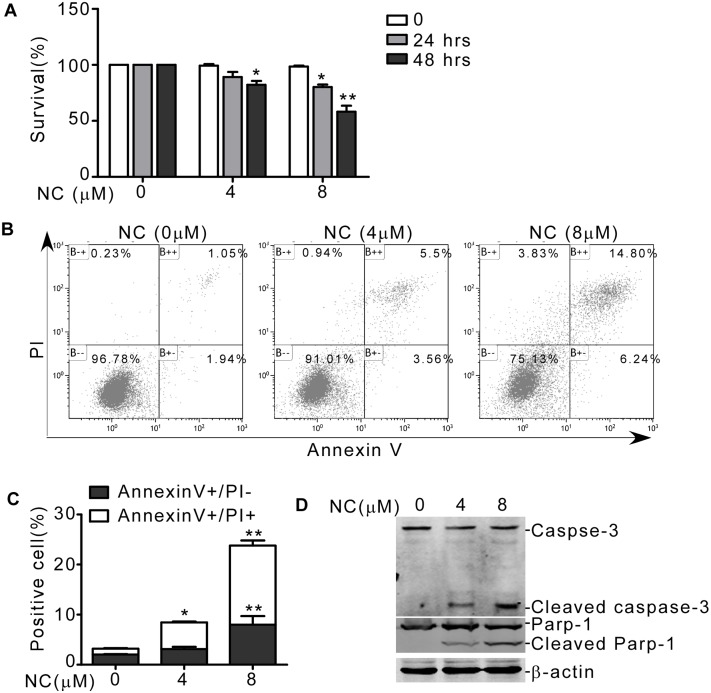
NC induced apoptosis in CML cells. (A) After K562 cells were treated with 4 or 8 μM NC for 24 or 48 hrs, MTT assay was performed to evaluate cell viability. The values represent the means ± S.E. (n = 3). *, *P* < 0.05. **, *P*<0.01. (B and C) After K562 cells were treated with 4 or 8 μM NC for 2 days, apoptosis rate was detected by annexin V/PI double staining. The values represent the means ± S.E. (n = 3). *, *P*< 0.05. **, *P*<0.01. (D) After K562 cells were treated with 4 or 8 μM NC for 2 days, cleaved caspese-3 and Parp-1 were detected by western blot.

It had been studied that NC caused apoptosis in tumor cells [15]. Here, we determined whether NC could suppress CML cell viability. As shown in Fig. 2A, NC treatment for 24 or 48 hrs remarkably resulted in decreased viability of K562 cells (Fig. 2A). To confirm whether NC induced cell viability reduction was due to apoptosis, annexin V/PI double staining was executed. Treatment with 4 and 8μM of NC for 48hrs resulted in significant higher apoptotic rate by 10.56 ± 1.47% and 23.78 ± 5.3%, respectively (Fig. 2B and C), suggesting that NC could induce apoptosis in CML cells. To further investigate the mechanisms involved in NC-mediated apoptosis, we measured caspase-3 activity. As shown in Fig. 2D, NC treatment significantly increased cleaved caspase-3 level as well as the cleavage of its substrate Parp-1. These results showed that NC could promote apoptosis by activating caspase-3.

### NC downregulated c-Myc protein level by accelerating its degradation in K562 cells

**Fig 3:**
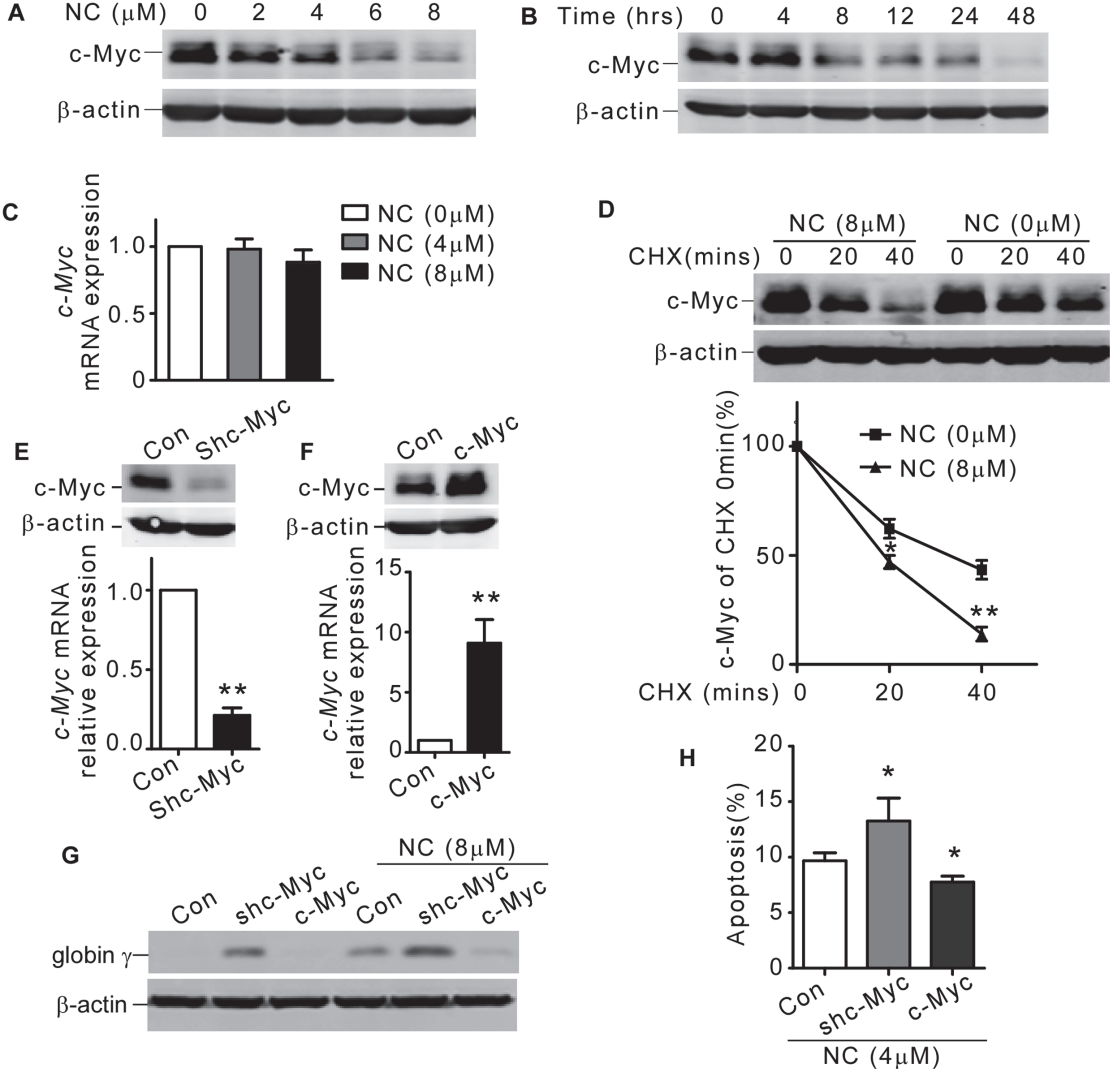
NC downregulated c-Myc protein level by accelerating degradation in K562 cells. (A and B) K562 cells were treated with 0, 2, 4, 6 or 8 μM NC for 2 days, or 8 μM NC for 0, 4, 8, 12, 24 and 48 hrs. Western blot analysis was performed to examine the expression level of c-Myc, and β-actin was used as internal control. (C) Real time qRT-PCR showed the *c-Myc* mRNA levels in K562 cells treated with indicated concentrations of NC for 2 days. *β-actin* was used as internal control. The values represent the means ± S.E. (n = 3). (D) K562 cells were exposed to 8 μM NC for 2 hrs, and then chased with 150 μg/mL CHX for 0, 20 and 40 mins. c-Myc and β-actin were detected by western blot. The values represent the means ± S.E. (n = 3). *, *P* < 0.05. **, *P*<0.01. (E) K562 cells were stably transfected with shc-Myc or control shRNA. (F) K562 cells were stably transfected with pIRES-c-Myc or control plasmid. c-Myc protein (upper panel) and mRNA (lower panel) level was detected by western blot and real-time qRT-PCR, respectively. The values represent the means ± S.E. (n = 3). **, *P*<0.01. (G) K562 cells stably transfected with shc-Myc, pIRES-c-Myc or control plasmid, were exposed to NC for 2 days. globin γ and β-actin were detected by western blot. (H) K562 cells stably transfected with shc-Myc, pIRES-c-Myc or control plasmid, were exposed to NC for 2 days. Apoptosis rate was detected by annexin V/PI double staining. The values represent the means ± S.E. (n = 3). *, *P* < 0.05.

The proto-oncogene c-Myc had been shown to play pivotal roles in cell differentiation and chemosensitivity in CML [7, 8]. To investigate whether NC could affect the expression of c-Myc, K562 cells were treated with NC and endogenous c-Myc was detected by western blot. As shown in Fig. 3A and B, endogenous c-Myc was downregulated in a NC dosage- or time-dependent manner. To dissect the detailed mechanism, we measured the mRNA level of *c-Myc* by real-time qRT-PCR in K562 cells. As shown in Fig. 3C, NC treatment had little effect on the mRNA level of *c-Myc*, suggesting that NC elicited c-Myc protein attenuation could be mediated on the post-translational level. c-Myc was reported to be mainly degraded through ubiquitin-proteasome pathway. To confirm whether NC could mediate c-Myc degradation, we determined the degradation kinetics of c-Myc by CHX chase assay after NC treatment. Western blot showed NC treatment could markedly accelerate the degradation of c-Myc (Fig. 3D), indicating that downregulation of c-Myc by NC was due to enhanced proteolysis.

### NC induced K562 differentiation and apoptosis were further enhanced by c-Myc interference, and reversed by c-Myc overexpression

To determine the functional role triggered by c-Myc in NC induced erythroid differentiation and apoptosis, we established K562 cell lines knocking down or overexpressing c-Myc (Fig. 3E and F). Globin γ was a common marker of erythroid differentiation. As shown in Fig. 3G, protein level of globin γ was elevated during NC induced erythroid differentiation (lane 1 and 4). On the other hand, globin γ was markedly decreased in c-Myc overexpressing cells (lane 3 and 6), while knocking down c-Myc increased globin γ protein level (lane 1 and 2), which was further enhanced by combination of c-Myc shRNA and NC treatment (lane 5). Next we studied the roles of c-Myc in NC mediated apoptosis by flow cytometry. We found that NC induced apoptosis was further triggered by c-Myc downregulation, while overexpressing c-Myc could partially rescued NC elicited viability decrement in K562 cells (Fig. 3H). These findings provided strong evidences that c-Myc was intimately involved in NC induced differentiation and apoptosis of K562 cells.

### NC accelerated c-Myc degradation via enhanced Thr58 phosphorylation

**Fig 4:**
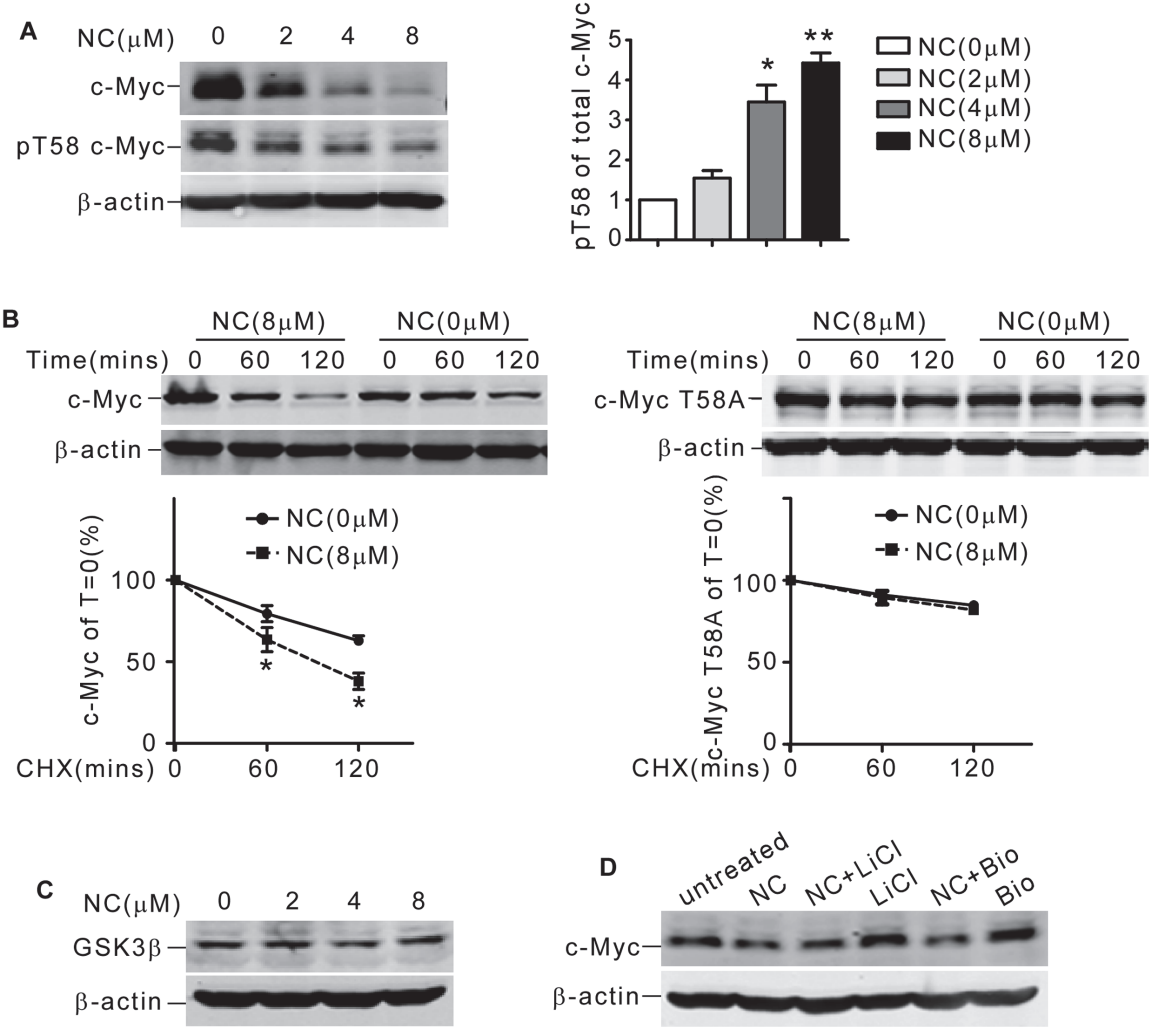
NC accelerated c-Myc degradation via enhanced Thr58 phosphorylation. (A) K562 cells were treated with 0, 2, 4 or 8μM NC for 2 days. c-Myc, phosphorylated c-Myc on Thr58 residue (pT58 c-Myc) and β-actin were detected by western blot. The values represent the means ± S.E. (n = 3). *, *P*< 0.05. **, *P*< 0.01. (B) After transfected with GFP-tagged wild-type (left panel) or c-Myc T58A mutant (right panel) plasmids, K562 cells were exposed to NC for 2hrs, then CHX chase assay was performed. c-Myc expression was detected by anti-GFP antibody. β-actin was used as loading control. The values represent the means ± S.E. (n = 3). *, *P*< 0.05. (C) K562 cells were treated with 0, 2, 4 or 8 μM NC for 2 days. GSK3β and β-actin levels were detected by western blot. (D) K562 cells were pretreated with GSK3β inhibitor LiCl (40 mM) or Bio (0.5 μM) for 2hrs, and then cells were exposed to 8 μM NC for 24 hrs. c-Myc and β-actin were detected by western blot.

As phosphorylation of c-Myc on Thr58 residue was decisive for its degradation [24], we asked whether NC could promote c-Myc Thr58 residue phosphorylation. To test this possibility, Thr58 phosphorylated c-Myc was detected after NC treatment. As shown in Fig. 4A, phosphorylated c-Myc at Thr58 was significantly increased normalized to total c-Myc. We then performed CHX chase assay to evaluate the half-life of GFP tagged wild type and T58A mutated (threonine to alanine) c-Myc. We found GFP tag slowed down c-Myc degradation (Fig. 4B, left) comparing to endogenous c-Myc (Fig. 3D), so we extended the time duration of CHX chase assay. Consistent with our previous results, NC could markedly enhance degradation of wild type c-Myc (Fig. 3D and 4B, left). However, NC failed to promote degradation of c-Myc T58A mutant (Fig. 4B, right). From these results, we concluded that phosphorylation of Thr58 residue was critical for NC induced c-Myc degradation.

It had been well demonstrated that GSK3β phosphorylated c-Myc at Thr58 and triggered its proteasomal degradation [25]. To examine whether GSK3β was involved in response to NC, we detected GSK3β protein level after NC treatment in K562 cells. We found the total proportion of GSK3β did not change significantly (Fig. 4C). Next we studied whether NC could influence GSK3β kinase activity. K562 cells were pretreated with GSK3β inhibitor LiCl or Bio before NC addition, and then cells were subjected for western blot analysis. We found LiCl or Bio rarely affected NC-induced c-Myc degradation (Fig. 4D), implying that NC could not affect kinase activity of GSK3β. Thus, we proposed that NC induced degradation of c-Myc could be independent of GSK3β.

### NC elicited erythroid differentiation and apoptosis were mediated by c-Myc- activated miRNAs

**Fig 5:**
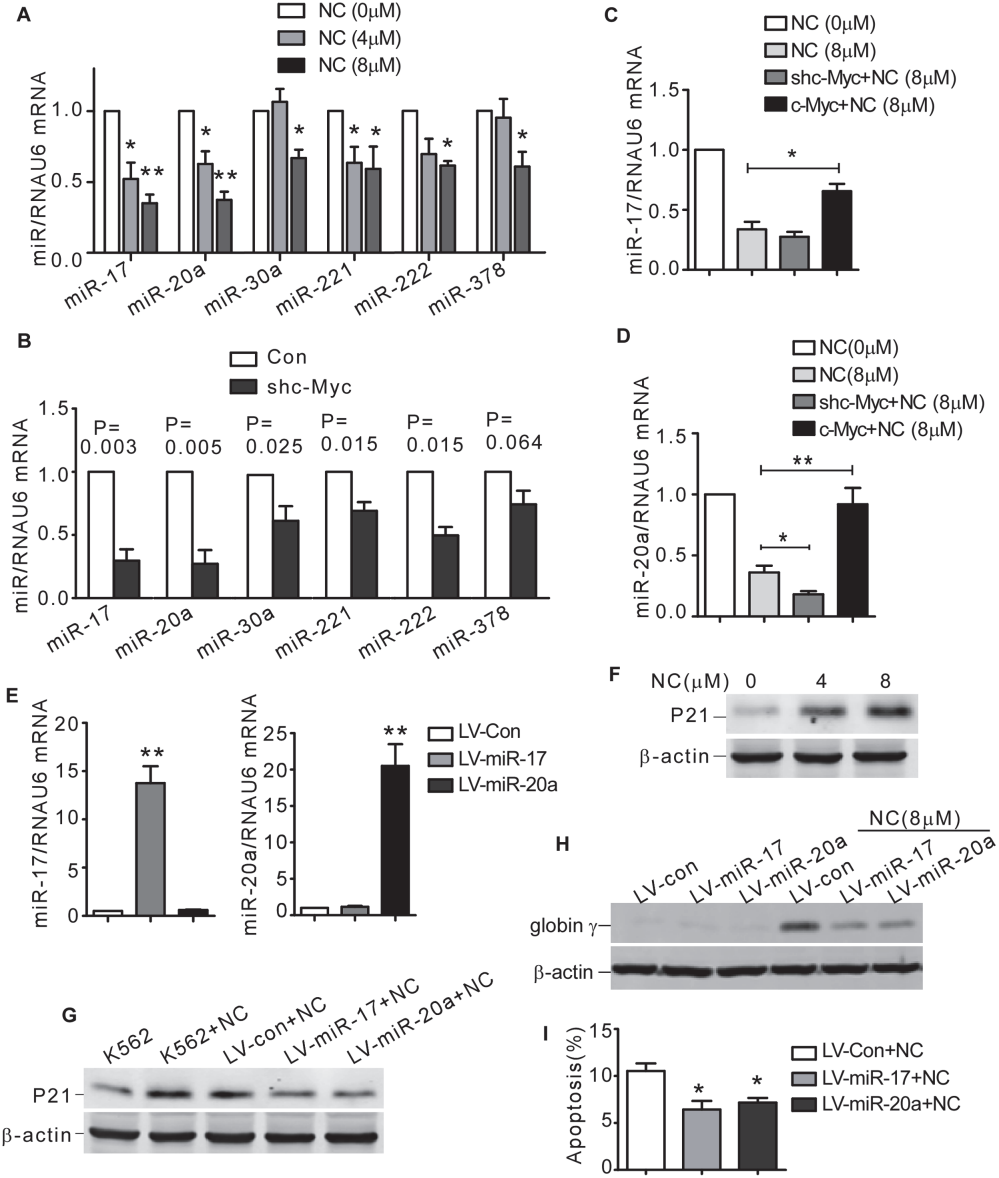
NC downregulated c-Myc activated miRNAs. (A) K562 cells were treated with 0, 4 or 8 μM NC for 2 days, the relative levels of mature miR-17, miR-20a, miR-30a, miR-221, miR-222 and miR-378 were detected by real-time qRT-PCR. The values represent the means ± S.E. (n = 3). *, *P*<0.05. **, *P*<0.01. (B) K562 cells were stably transfected with shc-Myc or control (Con). The relative expression of miR-17, miR-20a, miR-30a, miR-221, miR-222 and miR-378 was detected by real-time qRT-PCR. The values represent the means ± S.E. (n = 3). (C and D) Stably transfected K562 cells with shc-Myc, pIRES-c-Myc or control plasmid were exposed to NC for 2 days. The relative expression of mature miR-17 (C), miR-20a (D) was detected by real-time qRT-PCR. The values represent the means ± S.E. (n = 3). *, *P*<0.05. **, *P*<0.01. (E) The K562 cells were infected with LV-miR-17, LV-miR-20a or control (LV-con) lentiviral particles for 3 days, and the relative level of mature miR-17 and miR-20a was detected by real-time qRT-PCR. The values represent the means ± S.E. (n = 3). **, *P*<0.01. (F) K562 cells were treated with 0, 4 or 8 μM NC for 2 days, and then p21 and β-actin were detected by western blot. (G and H) After infection with LV-miR-17, LV-miR-20a or control lentiviral particles for 3 days, K562 cells were treated with NC (8 μM) for additional 2 days. p21 and globin γ were detected by western blot. β-actin was used as loading control. (I) After infection with LV-miR-17, LV-miR-20a or control lentiviral particles for 3 days, K562 cells were treated with NC (4 μM) for additional 2 days. Apoptosis rate was detected by annexin V/PI double staining. The values represent the means ± S.E. (n = 3). *, *P*<0.05.

A specific group of miRNAs, which had been reported to be induced in response to c-Myc activation, mediated biological functions of c-Myc [26, 27]. We next examined the effects of NC on the expression of c-Myc activated miRNAs (miR-17, miR-20a, miR-30a, miR-221, miR-222 and miR-378), which were typically increased in leukemia and triggered to the development of leukemia [11, 20, 21, 22]. Our results revealed that NC treatment decreased the relative levels of miR-17, miR-20a, miR-30a, miR-221, miR-222 and miR-378, among which miR-17 and miR-20a showed the sharpest decrement by 65.0 ± 0.6% and 62.6 ± 2.6%, respectively (Fig. 5A). These data suggested that NC could regulate the expression of c-Myc associated miRNAs in CML cells.

We next explored the effects of c-Myc inactivation on the expression of the tumor associated miRNAs (miR-17, miR-20a, miR-30a, miR-221, miR-222 and miR-378) in K562 cells. We found that c-Myc downregulation led to decreased expression of miR-17, miR-20a, miR-30a, miR-221, miR-222 and miR-378 (Fig. 5B), which were consistent with the effects of NC shown in Fig. 5A.

To further confirm whether impaired c-Myc expression was responsible for the decreased expression of miR-17 and miR-20 upon NC treatment, we detected the expression of miR-17 and miR-20a in K562 stably overexpressing c-Myc. Our results showed that continuous overexpression of c-Myc significantly reversed miR-17 and miR-20a expression in K562 cells exposed to NC (Fig. 5C and D). These results suggested downregulation of c-Myc was responsible for NC induced decrease of miR-17 and miR-20a.

### MiR-17 and miR-20a antagonized NC-induced differentiation and apoptosis of K562 cells

To investigate whether overexpression of miR-17 or miR-20a would influence the ability of NC to induce erythroid differentiation, we constructed lentiviral vectors harboring pri-miR-17 or pri-miR-20a sequence. Infection of K562 cells with LV-miR-17 or LV-miR-20a lentiviral particles caused a significant upregulation of mature miR-17 or miR-20a level (Fig. 5E). p21 was reported to be one of the target genes of miR-17 and miR-20a [28]. We found NC could markedly upregulate p21 (Fig. 5F), whereas overexpression of miR-17 and miR-20a antognized the protein level increment of p21 in K562 cells after treatment with NC (Fig. 5G). Previous studies showed that p21 could be modulated by both c-Myc and members of miR-17 family [29, 30]. We antagonized the expression of miR-17/20a by transfection of their specific antisense oligonucleotides (AS) or negative control (scramble oligonucleotides, SC) prior to NC treatment in K562 stably overexpressing c-Myc, and found both c-Myc and miR-17/20a were involved in upregulation of p21 induced by NC (S1 Fig.). Furthermore, we detected the effect of miR-17 or miR-20a on the expression of globin γ with or without NC treatment in K562 cells. We found that NC treatment could markedly improve globin γ expression, which was accordant with our previous studies (Fig. 1D), while miR-17 or miR-20a could remarkably weaken the incremental effects of NC on globin γ (Fig. 5H, right 3 lanes). By flow cytometry, we found overexpression of miR-17 or miR-20a reversed apoptosis induced by NC (Fig. 5I). Taken together, these results suggested that c-Myc-activated miR-17/20a was involved in the erythroid differentiation and apoptosis induced by NC in CML cells.

### NC enhanced the biological effect of imatinib in K562 and primary CML cells

**Fig 6:**
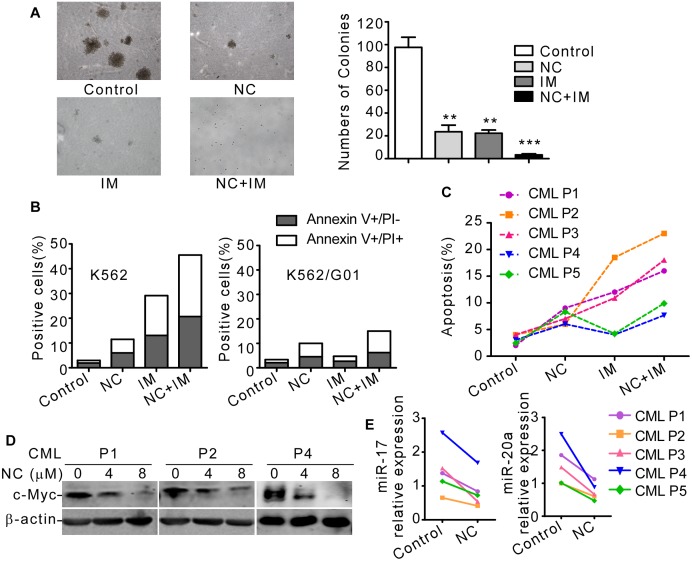
NC enhanced the biological effects of imatinib in K562 and primary CML cells. (A) Soft agar colony formation assay was performed in K562 cells after treatment with NC (4 μM), IM (0.2 μM) or both. Colonies >0.1 mm in diameter were counted under a microscopic bright field at 50× magnification. One representative picture was shown. Columns represented means ± S.E. (n = 3). **, *P*<0.01. ***, *P*<0.001. (B) Apoptosis rate was detected by annexin V/PI double staining after K562 or K562/G01 cells were treated with NC (4 μM), IM (0.2 μM) or NC+IM for 48 hrs. (C) BMMCs from 5 CML patients were treated with NC (4 μM), IM (0.5 μM) or NC+IM for 48 hrs, and annexin V/PI double staining was used to detect apoptotic rate. (D) BMMCs from three CML patients (P1, P2 and P4) were treated with 0, 4 or 8 μM NC for 2 days. c-Myc and β-actin were detected by western blot. (E) BMMCs from five CML patients were treated with 8 μM NC or control for 2 days. The relative level of mature miR-17 and miR-20a were detected by real-time qRT-PCR. RNU6 was used as internal control.

Imatinib (IM), one of tyrosine kinase inhibitors (TKIs), specifically inhibiting BCR-ABL fusion protein and triggering apoptosis and differentiation of CML cells, is used as first-line treatment for CML. However, the resistance to IM is a growing obstacle for CML therapy [3]. c-Myc was considered to be a downstream executor of BCR-ABL [5], and c-Myc overexpression contributed to CML drug resistance [8, 9]. To investigate whether NC enhanced the inhibitory functions of IM, we firstly studied the colony growth of K562 cells after treated with NC or IM. Fig. 6A showed that both NC and IM caused significantly inhibition of colony growth, and co-treatment with NC and IM resulted in the slowest colony growth comparing with either agent alone. Next, we determined the effect of NC on apoptosis by flow cytometric analysis. K562 cells co-treated with NC and IM showed higher percentage of apoptosis (44.83 ± 5.65%) than cells treated with either NC (10.56 ± 1.47%) or IM (29.49 ± 2.25%). More importantly, NC could also induce apoptosis (9.89 ± 1.38%) of K562/G01, an IM resistant cell line [31] (Fig. 6B), similar to that of K562 cells. K562 or K562/G01 cells were treated with NC or IM of various concentrations for 48 hrs and cell proliferation was measured by MTT assay. Unlike IM, which failed to inhibit viability of K562/G01 cells (S2A Fig.), NC decreased survival ability of both cell lines (S2B Fig.). We next detected the anti-leukemia effect of NC and/or IM in primary CML cells isolated from bone marrow of 5 patients in the chronic phase of CML. All 5 patients were newly diagnosed, and none of them were previously treated with any TKIs. The CML patient 1, 2 and 3 were subsequently proved to be IM responders, as they achieved complete hematologic remission within three months and major cytogenetic remission within 12 months, based on the European Leukemia Net treatment guidelines. Conversely, patient 4 and 5 were IM nonresponders, and they did not succeed to achieve major cytogenetic remission within 12 months of IM treatment. Bone marrow mononuclear cells (BMMCs) from 5 CML patients were treated with NC and/or IM, and flow cytometry was performed to detect the apoptosis of primary CML cells. We found two patients (sample 4 and sample 5) showed IM resistance, but interestingly, NC could still induce apoptosis in samples of patient 4 and 5 (Fig. 6C). Next we found that the c-Myc protein level (Fig. 6D) and miR-17/20a (Fig. 6E) level were decreased after NC treatment in primary CML cells, illustrating that inhibition of c-Myc and c-Myc activated miRNAs was involved in the biological effects of NC. ALL these results indicated that NC enhanced the effects of IM in K562 and primary CML cells, and both IM sensitive and resistant CML cells exhibited high sensitivity to NC.

## Discussion

CML is a clonal myeloproliferative syndrome, leading to increased production of granulocytes at all stages of differentiation [1]. TKIs rarely completely cured CML due to drug resistance and leukemia stem cells [32]. Differentiation-inducing therapy for leukemia is a new field in the research of leukemia treatment. Apparently, any attempt to discover differentiation inducers and understand the molecular mechanisms involved in leukemia cell differentiation could be helpful to improve therapeutic strategies.

Nitidine chloride (NC), first derived from *Zanthoxylum nitidum*, was a natural phytochemical alkaloid. It had been found that NC exhibited several types of biological activity, including anti-inflammation [33], anti-malaria [34], anti-fungi [35] and anti-angiogenesis [19]. Accumulating *in vitro* and *in vivo* studies showed that NC could exert its anti-tumor effect in a variety of malignancies by inducing apoptosis and cell cycle arrest [13, 14, 15, 16, 17, 18, 19]. In this study, we showed that NC exhibited strong effects to induce erythroid differentiation and apoptosis of K562 cells (Figs. 1 and 2). Erythroid differentiation and apoptosis were two independent processes that could be easily associated in leukemia. Some drugs or genes were involved in both differentiation and apoptosis, which could be mutually reinforced [36, 37]. To elucidate the molecular mechanisms of NC induced differentiation and apoptosis, we investigated the expression of c-Myc, which was dysregulated in leukemias, leading to differentiation inhibition and abnormal proliferation [38, 39, 40]. We found that NC downregulated c-Myc protein level in K562 cells.

Many strategies are currently under development to target c-Myc, such as transcriptional disruption (antisense oligonucleotides, peptide nucleic acids and small interfering RNA) or functional interuption by inhibiting critical protein-protein interactions [41, 42]. In this study, we found that NC downregulated c-Myc protein level in K562 cells by accelerating its degradation. The degradation of c-Myc through ubiquitin-proteasome pathway was triggered by GSK3β mediated phosphorylation at Thr58 residue [24]. However, we did not find GSK3β was involved in NC elicited c-Myc degradation, suggesting NC could function through other molecular mechanisms. Studies had shown that other factors could be directly or indirectly responsible for c-Myc protein stability [25, 43, 44, 45, 46] independent of GSK3β, implying that GSK3β was not the unique kinase to phosphorylate c-Myc at Thr58. Whether c-Myc degradation induced by NC functioned through these factors was still needed to be elucidated.

We suspected that NC elicited degradation disrupted biological activities of c-Myc and eventually resulted in erythroid differentiation and apoptosis in K562 cells. This notion was supported by the fact that overexpression of c-Myc partially abrogated differentiation and apoptosis induced by NC in K562 cells. However, the mechanisms by which c-Myc blocked differentiation and apoptosis were poorly understood. In our study, we found c-Myc-activated miRNAs were also involved in regulating erythroid differentiation and apoptosis induced by NC. miRNAs are short, non-coding RNAs that recognize target sequences of imperfect complementarity in cognate mRNAs, resulting in either translational repression or mRNA degradation [47]. A specific group of miRNAs, which had been reported to be induced in response to c-Myc, were thought to act as executors of c-Myc signaling pathway [26]. The expression of miR-17, miR-20a, miR-30a, miR-221, miR-222 and miR-378, which were reported to be dependent on c-Myc transcriptional activity [27, 48, 49, 50] and contribute the development of leukemia [11, 20, 21, 22], was examined in K562 cells treated with NC. We found that most of them, especially miR-17 and miR-20a showed significant downregulation after NC treatment.

miR-17 and miR-20a are two representative members of a highly conserved gene cluster miR-17–92, a miRNA polycistron also known as oncomir, and might have parallel roles through regulating the same target genes as they contain the same seed sequence [51]. Expression profiling had shown that miR-17 and miR-20a were overexpressed in varieties of solid tumors and hematopoietic malignancies, including MLL-rearranged leukemia [52], T-cell acute lymphoblastic leukemia [28, 53] and B-cell lymphoma [54, 55]. miR-17–92 cluster was frequently amplified or overexpressed in CML CD34^+^ cells and abnormal expression of miR-17–92 greatly increased proliferation of CML stem cells [11]. In our study, we found miR-17 and miR-20a were downregulated in the process of erythroid differentiation and apoptosis induced by NC in K562 cells. miR-17 and miR-20a overexpression partly attenuated NC-induced differentiation and apoptosis, suggesting that miR-17 and miR-20a might take part in tumorigenesis of CML. Members of the miR-17–92 cluster targeted numerous cancer suppressor genes, e.g. Pten [55], BIM [56], E2F1 [57], p21 [28] and STAT3 [58], showing functions in differentiation and apoptosis. It was reported that mutational loss of Pten elicited resistance to apoptosis induced by gamma-secretase inhibitors in T-cell leukemia [59]. p21 was closely correlated with differentiation in U937 cells, which occured independently of p53 [60]. BIM was reported to be a suppressor of c-Myc induced mouse B cell leukemia [61] and was also involved in differentiation induced by 1,25-Dihydroxyvitamin D3 in human myeloid leukemia cells [62]. Stat3 promoted the EPO induced erythroid differentiation of UT-7/GM cells [63]. These evidences supported that NC elicited erythroid differentiation and apoptosis were regulated by the biological effects of c-Myc-activated miRNAs, particularly miR-17 and miR-20a.

IM, used as first-line treatment for CML, is extraordinarily effective on patients in the chronic phase of CML. However, the resistance to IM was the biggest barrier for CML therapy [3]. The mechanisms of IM resistance included BCR-ABL gene mutations, BCR-ABL gene amplification and activation of other survival pathways, such as Src tyrosine kinase, ERK, PI3K, MDR1 and COX2 [64]. Liu *et al.* found c-Myc expression was upregulated in IM resistant CML cells and knockdown of c-Myc could sensitize IM resistant cells to apoptosis [65]. Porro *et al.* reported that c-Myc dictated transcriptional profiles of ATP-binding cassette (ABC) transporter genes in CD34^+^ CML progenitor cells, resulting in multidrug resistance [12]. These results indicated critical roles of c-Myc in chemosensitivity of CML. We found NC downregulated c-Myc protein level in K562 and primary CML cells, so we asked whether NC enhanced the effect of IM in CML cells. Here we found in IM sensitive K562 and primary CML cells, co-treatment of IM and NC showed higher apoptotic rate than that of either agent alone. Moreover, even IM resistant CML cells exhibited high sensitivity to NC. The remarkable efficiency of NC to induce cell apoptosis in K562/G01 (IM resistant CML cell line) and primary CML cells provided potential possibility to overcome IM resistance.

## Supporting Information

S1 Fig Downregulation of miR-17/20a reversed c-Myc mediated abrogation of p21 in the present of NC. (DOC)

S2 Fig NC induced loss of cell viability in K562 and K562/G01. (DOC)
