# Supplementary material for: Novel Agent Nitidine Chloride Induces Erythroid Differentiation and Apoptosis in CML Cells through c-Myc-miRNAs Axis
Source: PLoS One. 2015 Feb 3;10(2):e0116880. doi: 10.1371/journal.pone.0116880 (PMC4315404; doi:10.1371/journal.pone.0116880)
Supplement: S1 Fig — (DOC) [file pone.0116880.s001.doc]

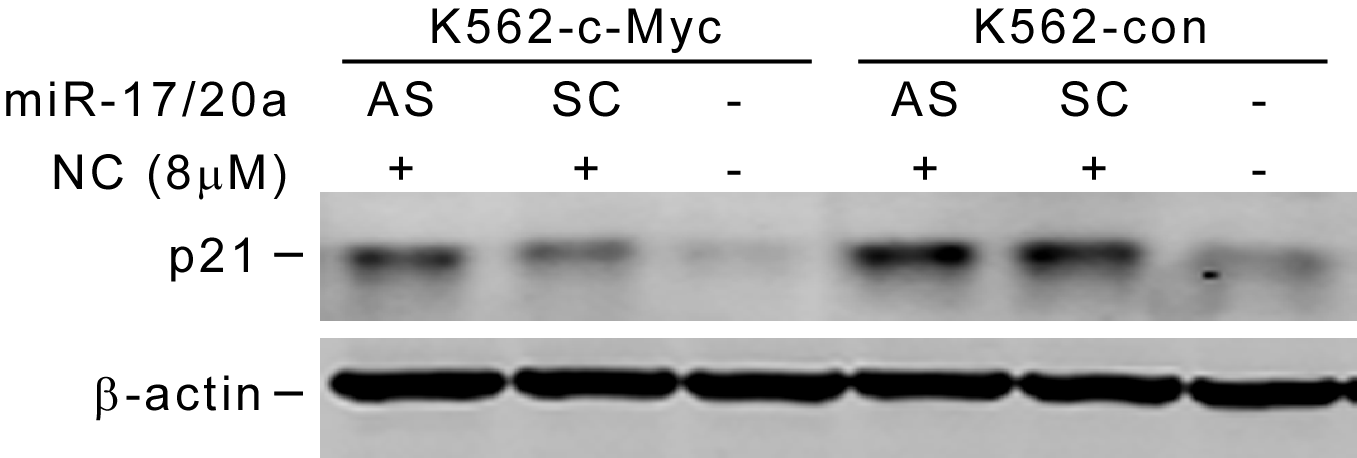


**Fig. S1 Downregulation of miR-17/20a reversed c-Myc mediated abrogation of p21 in the present of NC.** K562 stably overexpressing c-Myc or control cells were transfected with miR-17 and miR-20a specific antisense oligonucleotides (AS) or negative control (scramble oligonucleotides, SC), and then cells were treated with 8 μM NC for 48hrs. p21 and β-actin protein expression were examined using Western blotting analysis.
