# Supplementary material for: Novel Agent Nitidine Chloride Induces Erythroid Differentiation and Apoptosis in CML Cells through c-Myc-miRNAs Axis
Source: PLoS One. 2015 Feb 3;10(2):e0116880. doi: 10.1371/journal.pone.0116880 (PMC4315404; doi:10.1371/journal.pone.0116880)
Supplement: S2 Fig — (DOC) [file pone.0116880.s002.doc]

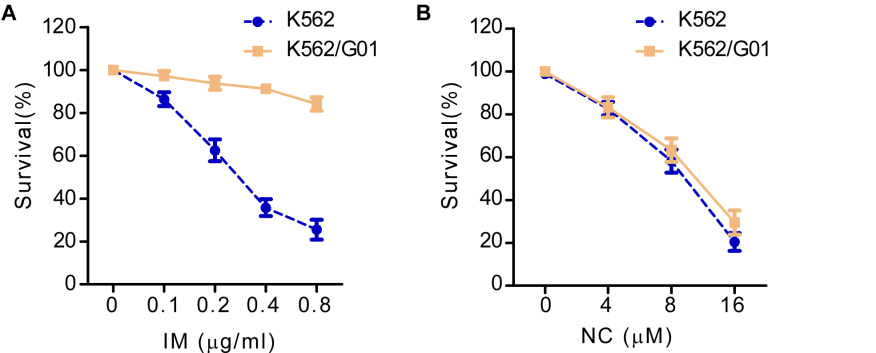


**Fig. S2 NC induced loss of cell viability in K562 and K562/G01.** K562 and IM-resistant K562/G01 were treated with IM (A) or NC (B) at various concentrations for 48 hrs and cell viability was measured using MTT assay. The values represent the means ± S.E. (n= 3).
